# Comparative Evaluation of Bond Strengths Between Dual Cure Resin Cement and Light Cure Resin Cement in Root Surface Indirect Restorations: An In Vitro Analysis Study

**DOI:** 10.7759/cureus.55009

**Published:** 2024-02-26

**Authors:** Karishma Desai, Karthickraj S M

**Affiliations:** 1 Department of Periodontics, Saveetha Dental College and Hospitals, Saveetha Institute of Medical and Technical Sciences, Saveetha University, Chennai, IND

**Keywords:** health, dental, luting, bond strength, veneers, resin cements, dual cure, light cure

## Abstract

Aim

The aim of this study was to evaluate the shear bond strength between dual cure and light cure resin cements in root surface indirect restorations.

Materials and methods

Ten recently extracted human teeth were selected. Cylindrical blocks of resin were prepared and bonded near the Cemento-Enamel Junction (CEJ) of the prepared teeth to mimic the restoration at the root surface. The samples were randomly luted to the root surface using the light cure (Calibra Veneer, Dentsply Sirona, India) and dual cure (Fusion Ultra D/C, DenPro, USA) forming two groups. The bond strength was checked using the INSTRON 3000 device (INSTRON, MA, USA). The point of fracture of the prepared sample from the tooth surface was noted. All readings were tabulated and further statistically analyzed.

Results

On comparing the two groups, it was found that the light cured resin had a greater mean shear bond strength (57 N) than the dual cure resin cement (41 N). The difference in the mean value of the shear bond strength between two resin cements was found to be statistically not significant according to independent T-test analysis using Levene’s Test (P>0.05).

Conclusion

From the results obtained and within the limits of the study conducted, we can infer that Calibra Veneer is a more viable option for luting to the root surface area. On the other hand, Fusion Ultra Dual cure resin cement seems to have similar results but has a lower bond strength than the other.

## Introduction

Patients are increasingly seeking treatment for unattractive anterior teeth. For many years, the preparation of full crowns has provided the most predictable and long-lasting aesthetic repair of anterior teeth. However, this procedure is unquestionably the most invasive, requiring the removal of significant volumes of sound tooth substance, which may have negative consequences on neighboring pulp and periodontal tissues [1-5]. Improvements in dentistry have expanded the treatment options for reconstructions, and direct or indirect resin composite restorations and ceramic veneers are now effective options to restore anterior teeth that have been lost to decay, trauma, or other reasons. A veneer is a thin porcelain shell that is bonded to the front of a tooth that has undergone only a little dental work using luting materials and dental adhesives. The ability of resin composites to adhere to the tooth is the foundation of this minimally invasive technique. For long-term retention of porcelain veneers, among other invasive treatment choices, a strong and stable link between the luting composite and the tooth is necessary [6-9].

Clinical tests show that the binding around porcelain veneer borders weakens, especially in the cervical areas of the anterior teeth. Not only may such debonding cause visual problems, but it could also cause secondary caries, post-operative sensitivity, and plaque development [10-12]. Furthermore, many patients seek treatment with veneers for periodontal diseases, which can lead to gingival recessions and exposed root surfaces in the area of the anterior teeth. Changes in tooth structure at various locations have a significant impact on the adhesion of resin-based products. For instance, the cervical enamel border, or CEJ, has a very different morphological structure from the incisal enamel [13-16]. In the cervical region, the enamel prism frequently has an ad hoc arrangement while having a more structured shape in the coronal region. The structural characteristics of cervical enamel affect how well restorative materials adhere to it. The CEJ is a complex region where various hard tissues of the tooth come together, including cementum overlapping enamel, cementum overlapping cementum, a gap exhibiting a strip of exposed dentin, and enamel overlying cementum. Both within and between teeth, the hard tissue distribution at the CEJ is variable and unpredictably distributed [17-20].

A crucial stage in assuring the durability of indirect restorations is bonding and cementation. Clinicians have access to a variety of dental luting agents, each of which has its own indications and methods. Because they are essentially insoluble and can survive the rigors of the oral environment, resin cements have outstanding mechanical qualities [21-23]. It is best to use light-curing cement when cementing porcelain veneers since resin cement can change color with time, becoming darker and less luminous even when it initially matches the substrate's hue. Although photo polymerisable, these cements have longer working times and better color stability than chemically cured and dual-cured cements; using them makes it easier to remove excess material before polymerization and shortens the time needed to finish the restoration after it has been cemented [24]. The root portion of the teeth consists of root cementum and root dentin onto which veneers are bonded in the tooth with receded gingiva, making it complicated for adhesive materials. The role of this study was to investigate the bond strength and evaluate the resin cement adhesion on the root surface. The aim of this study was to compare and evaluate the shear bond strength between light cure and dual cure resin cements at the root surface indirect restorations.

## Materials and methods

**Figure 1:**
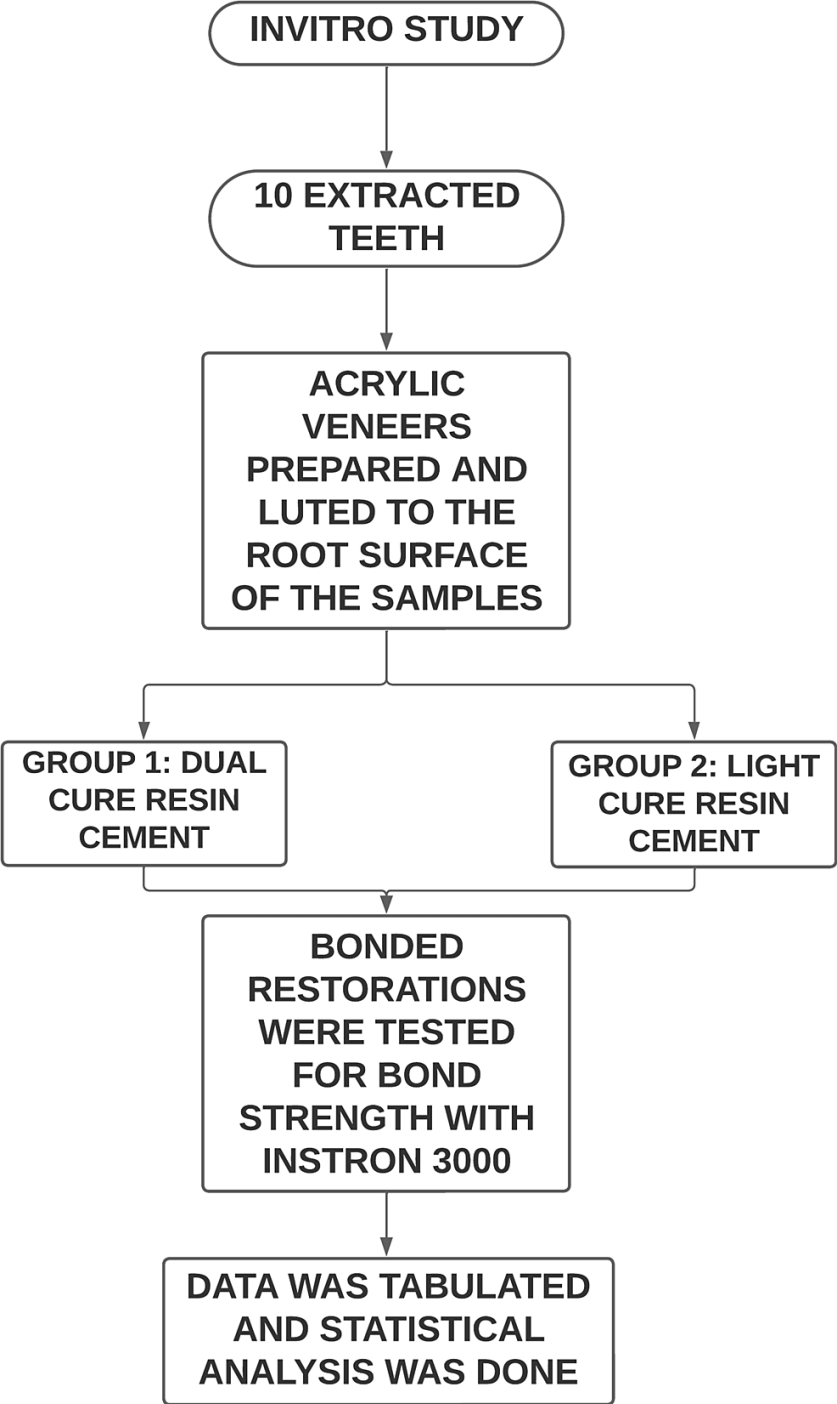
Study design flowchart

This in vitro study was designed to evaluate the bonding of the veneers to the root surface with dual cure and light cure resin cements (Figure 1). This study was performed at Saveetha Dental College, Chennai. The research protocol was approved by the Scientific Review Board at Saveetha Dental College (SRB/SDC/PERIO-1802/22/071).

Sample preparation

**Figure 2:**
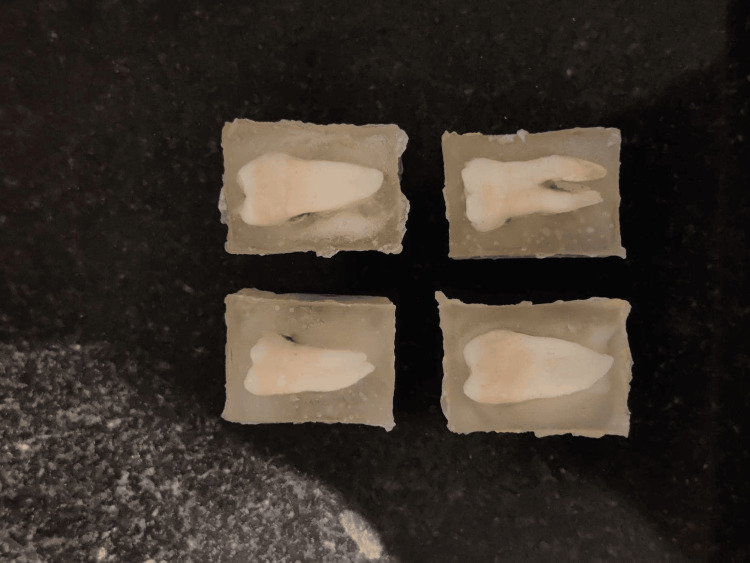
Preparation of the samples embedded in acrylic blocks Standard blocks of equal measurements were chosen. These samples were divided into two groups to carry out the study.

For this investigation, 10 posterior teeth were chosen. The following criteria were used to select the samples. Inclusion criteria were periodontally damaged and recently extracted posterior teeth. Exclusion criteria were non carious cervical lesions, fractures, decay, craze lines, previous restorations, abrasions, structural deformities, or any other fractures. Pre treatment of the tooth was done by calculus and debris removal, this was done by ultrasonic scaling. After treating the samples, they were stored in a medium containing normal saline. Saline is known to be safe as a storage medium as it is not known to cause any harmful effects to the tooth surface by causing any erosions or crazing, in addition to this, it does not interfere with the bond strength of the tooth. For better control in sample preparation, the tooth surface was embedded into standard molds of equal dimensions, parallel to the base and in equal depths. This is shown in Figure 2. 

Use of resin cement to bond veneer samples to the tooth

**Figure 3:**
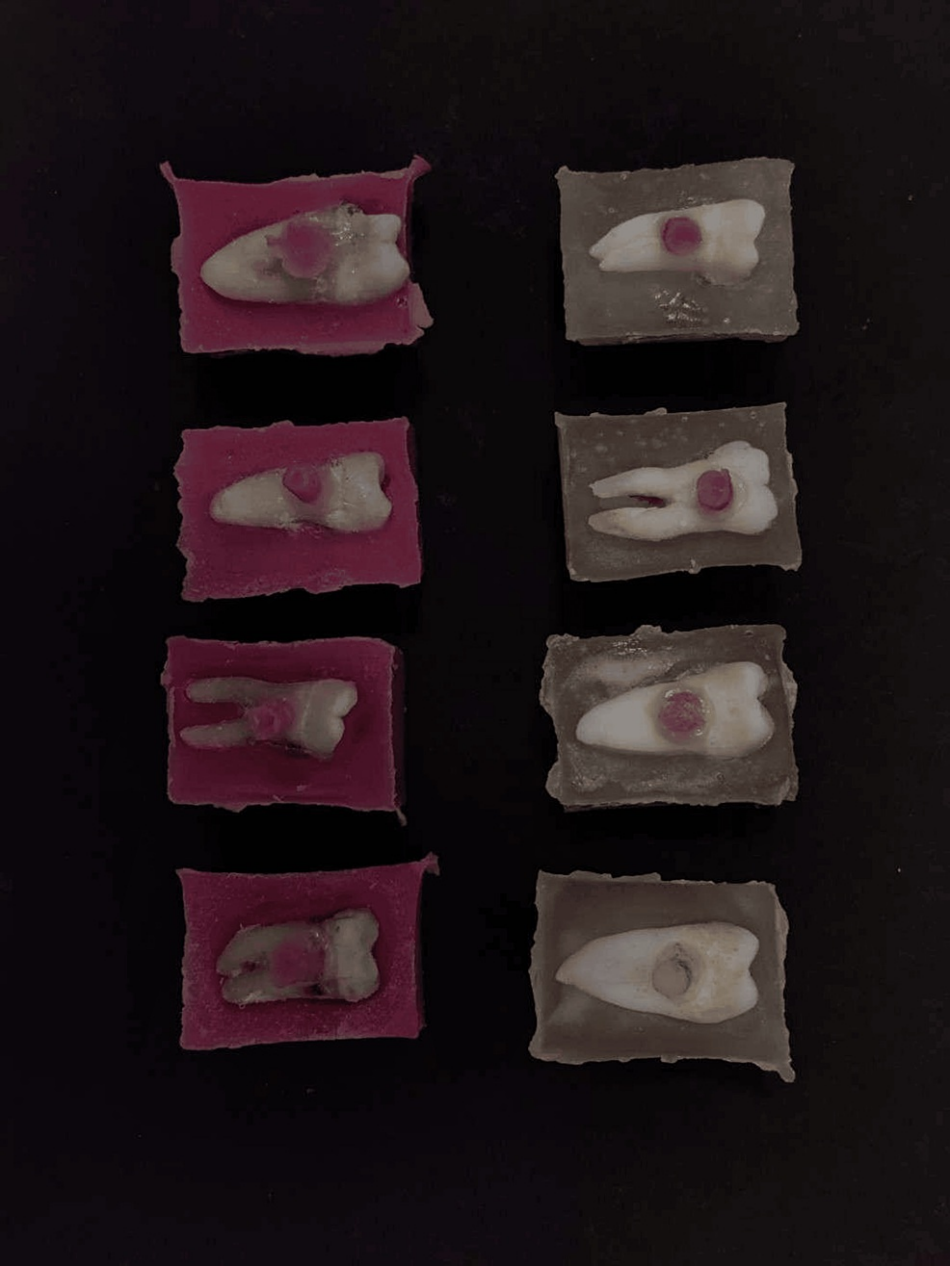
Veneer samples after bonding to the root surface The cylindrical veneer samples were bonded using Fusion Ultra D/C cement (Group 1) (Prevest DenPro, USA) and Calibra Veneer cement (Group 2) (Dentsply Sirona, India) which are dual curable and light curable, respectively.

After the tooth samples were prepared uniformly, smaller cylindrical samples were fabricated out of acrylic to form and mimic a veneer. These samples were randomly divided into two groups. The two groups of resin cements were investigated with five teeth each. These prepared samples were bonded to the root surface approximating the Cemento-Enamel Junction (CEJ) using two groups of resins were used for bonding - one group was using dual cure resin (Fusion Ultra D/C, DenPro, USA) and the other group was using light cured resin (Calibra veneer, Dentsply Sirona, India). The following bonding protocols were followed. 37% phosphoric acid was used to etch the tooth surface for 20 seconds. After rinsing with water, the surface was blot dried. After applying for 20 seconds, the self-priming bonding agent is light-cured for an additional 20 seconds. The respective resin cements were placed onto the veneer samples and pressed against the prepared root surfaces and light polymerisation was done before removing the excess cement residues from the tooth surface (Figure 3). The bonded samples were then kept for a full day, at 37°C ± 2°C, in distilled water prior to the shear bond strength test.

Evaluation of bond strength

**Figure 4:**
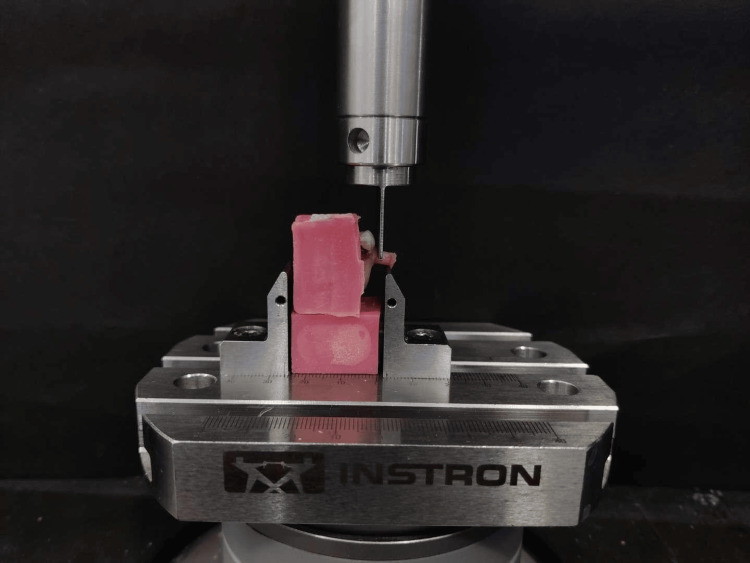
Evaluation of bond strength using INSTRON 3000 device

To evaluate the shear bond strengths of the resin cements, the samples were placed perpendicularly in the INSTRON 3000 machine and all specimens were loaded to failure (Figure 4). Bond strength was evaluated in Newton Units. Data for each sample was tabulated and mean values of shear bond strength were analyzed to find differences between the means with a 0.05 confidence level. The Fusngths were compared using the Student's t-test (α = 0.05). The statistical program Statistical Package for the Social Sciences (SPSS), version 24.0 (IBM Corp., Armonk, NY) was used to conduct the statistical analyses.

## Results

**Figure 5:**
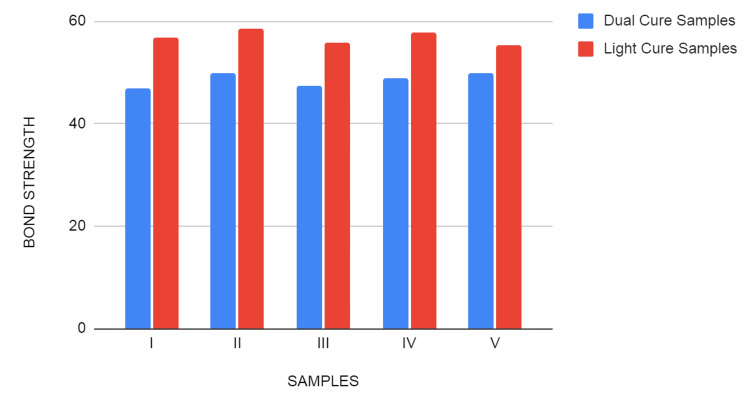
Comparison of shear bond strength between light cure and dual cure resin cements

The shear bond strength of Calibra veneer was found to be higher than that of the dual cure fusion ultra cement for every sample (Figure 5). The mean shear bond strength of dual cure samples and light cure samples were 48.568 and 56.846 respectively (Table 1). The difference in the mean value of the shear bond strength between two resin cements was found to be statistically not significant according to independent T-test analysis using Levene’s Test (P>0.05).

**Table 1:** The mean value of shear bond strength for the dual cure and light cure samples The dual cure and light cure samples that were tested and statistically not significant with independent T-test analysis (p=0.8). The value of the bond strength is expressed in Newton Units, F is the test statistic of Levene's test and Sig. is the p-value corresponding to this test statistic.

Groups	N	Mean	F	Sig.
Dual cure samples	5	48.568	0.068	0.8
Light cure samples	5	56.846		

**Figure 6:**
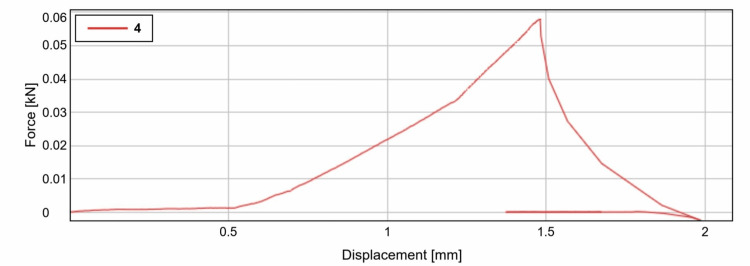
Graphical representation of the break point of Group 2 samples

**Figure 7:**
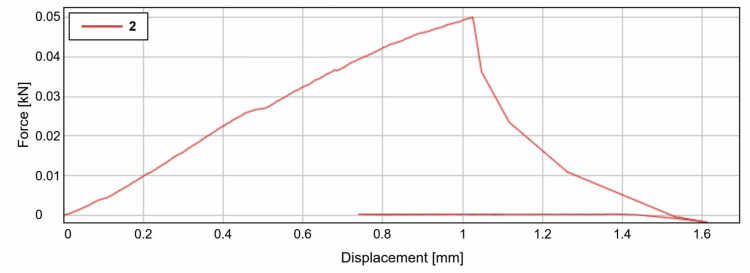
Graphical representation of the break point of Group 1 samples

As per the study, we can observe clearly that the shear bond strength of the light cured resin was an average of 56.8 N (Calibra Veneer, Dentsply Sirona, India) when it showed failure with the displacement of around 1.5mm (Figure 6) and was found to be comparatively higher than that of the dual cured resin cement which was at an average of 48.5 N (Fusion Ultra D/C, DenPro, USA) when it showed failure with the displacement of around 1mm (Figure 7).

## Discussion

The current study investigated the shear bond strength of the dual cure and light cure resin cements against the root surfaces of the tooth. There was no statistical difference in the independent T-test analysis using levene’s test (p=0.8) concluding that the difference in the mean values of shear bond strength between Group 1 (48.568) and Group 2 (56.846) was not substantial enough to accept that Group 2 resin cement was comparatively efficient bond strength to the root surfaces.

In a study by Andermatt and Ozcan [6], adhesion to root cementum yielded the lowest results, regardless of the adhesive technique used. The self-etch method had less favorable outcomes on dentin than the etch and rinse method, even though adherence to the cementoenemal junction was reduced. When compared to the self-etch system, the etch-and-rinse adhesive system showed greater micro-shear bond test (µSBT) values for dentin and root cementum. Findings from this study showed that cementum's organic structure hindered the bonding of the restorations with lower bond strength.

A study by Abu Haimed et al. [12], stated that there was a statistical difference in the bond strength with different resin cements against the pressable lithium disilicate glass ceramics regardless of the number of pressing cycles. The type of resin cements affected the shear bond strength of the pressable ceramics, which is in contradiction to the current study where there was no statistically significant difference found between the bond strength of the two groups. Another study by Lise et al. [13] showed that the mean Shear Bond Strength values of Variolink II were greater than the RelyX Unicem 2. This difference is also in contrast to the current study. Aside from the bond strength of the luting cement to the restoration and the tooth surface, another crucial issue to consider is the film thickness. These cements lack the wear resistance of the repair, and the bond strength may be compromised if the coating is either thin or too thick

The findings observed in a study done by Geeta et al. [25] in 2013 showed that the shear bond strength of dual cure composite resin is comparatively higher than that of the conventional light cure resins in the majority of the samples. This is contradictory to the current study as it shows a higher shear bond strength during the use of conventional light cure resin cements. This can be due to external factors affecting the study, like study setup, bonding technique, time of curing, and brand of the cement used is also a factor that can majorly affect the results obtained.

A study by Taher and Hamza [26] compared the shear bond strength of cementum and cervical dentin with resin composite using a universal adhesive system and resin-modified glass ionomer found that there was better bond strength with resin-modified glass ionomer cement rather than resin composites with universal adhesives. Toldeno et al. conducted a study to compare and analyse the bond strength of various adhesives and nanoroughness on pretreated cementum surfaces and found that an etch-rinse single bond had comparatively higher bond strength on cementum surfaces than the other adhesives. The higher bond strength of the cementum surface was observed because of the increased surface bonding area with etchant and resin infiltration from the adhesives enhancing the bond strength of the cementum surfaces [27].

As per a study done by Deniz et al. [28] in 2000, in comparison to Super-Bond cement, Panavia-Ex cement demonstrated greater bond strength. The mechanical qualities of Panavia-Ex cement and the ceramic oxide and ester bond were credited with this increased bond strength. This shows that resin luting cements showed a higher shear bond strength and a better postoperative result. Another study done by Aksornmuang et al. [29] in 2006 showed that there were no statistical differences between the shear bond strength of light cured cement and dual cure resin cement; however, the post operative application and curing of silane couple agent showed a significant enhancement in the bond strength of the cement. In a study by Gundogdu and Aladag [30], Shear bond strength evaluation was done with a universal testing machine to evaluate the self-etch adhesive and self-adhesive resin cements, which showed that the former exhibited better shear strength than the latter. When compared to the current study, dual cure and light cure resin cements were only used, and self-adhesive cements were not incorporated in the current study. From the results obtained and within the limits of the study conducted, we can infer that Calibra Veneer is a more viable option for luting to the root surface area. On the other hand, Fusion Ultra Dual cure resin cement seems to have similar results but has a lower bond strength than the cement discussed above.

Limitations of the study

When compared to a clinical setting, the current investigation had certain drawbacks. The samples taken for this in vitro study were limited, and it could be increased in future prospective studies. Resin cements investigated in this study were also limited and different resin cements available would be included to improve in the further studies with more variables. Prior to the shear bond strength test, the specimens were first short-term kept for 24 hours at 37°C ± 2°C in distilled water. The in vitro nature of this experiment may not accurately imitate the changes in the bond strength and degradation of adhesive resin cements during extended periods of time under oral fluid circumstances, since the effects of thermocycling and long-term storage were not evaluated. Furthermore, in vitro investigations cannot replicate the static, chemical, and cyclic mechanical fatigue processes of the interfaces between the ceramic material and the tooth structure. Further investigation into these occurrences is necessary for replicating the clinical scenario and should be prioritized in future in vitro investigations.

## Conclusions

From the results obtained and within the limits of the study conducted, we can infer that Calibra Veneer is a more viable option for luting to the root surface area. On the other hand, Fusion Ultra Dual cure resin cement seems to be having similar results but comparatively has a lower bond strength. It has also been observed that conventional light curable resin cements, like Calibra veneer, are more functional, seem to have enhanced properties, had higher shear bond strength. Other studies have shown that this cement even has a lower tendency of causing microleakage which leads to failure of the restorative prosthesis. Further studies are needed with a larger sample size and a wider range of resin cements for better clinical implications and recommendations.
